# Serum omentin-1 level in patients with benign prostatic hyperplasia

**DOI:** 10.1186/s12894-020-00623-4

**Published:** 2020-05-06

**Authors:** Haiqing He, Shuiqing Wu, Jun Hao, Long Wang, Kai Ai, Xuan Zhu, Ran Xu, Xiaokun Zhao, Yinhuai Wang, Zhaohui Zhong

**Affiliations:** 1 Department of Urology, The Second Xiangya Hospital, Central South University, Changsha, 410011 Hunan China; 2 Vancouver Prostate Centre, Department of Urologic Sciences, Faculty of Medicine, University of British Columbia, Vancouver, BC Canada; 3 Department of Urology, Xiangya Hospital, Central South University, Changsha, 410008 Hunan China

**Keywords:** Benign prostatic hyperplasia (BPH), Omentin/Omentin-1, Adipokine, Prostate volume, Interleukin-8, Interleukin-18

## Abstract

**Backgroud:**

To evaluate the relationship between omentin-1 and benign prostatic hyperplasia (BPH). BPH is the most common urological disease in elderly men worldwide. Lower serum omentin-1 levels were reported to be negatively associated with the incidence of inflammation, diabetes, obesity and metabolic syndrome, which all play a role in the development of BPH. To the best of our knowledge, the relationship between omentin-1 and BPH has not been investigated previously.

**Methods:**

A total of 70 males participated in this study, including forty patients diagnosed with BPH and thirty healthy males. The anthropometric measurements and the biochemical parameters were measured in this study. We evaluated serum omentin-1 levels and the correlation with those data. We also test the gene expression of IL-8, IL-18 in BPH group using the TURP tissues.

**Results:**

The serum omentin-1 levels were lower in the BPH patients than in the control group (27.95 ± 4.18 versus 32.03 ± 5.46, *p* < 0.001). The general characteristics and biochemical parameters were investigated, and a negative correlation was found between serum omentin-1 levels and BMI in the BPH group (r = − 0.391, *p* = 0.013) as well as the whole group (r = − 0.457, p < 0.001). Multiple-factor binary regression analysis revealed that serum omentin-1was a protective factor of BPH development. Furthermore, lower serum omentin-1 levels were associated with higher mRNA expression of IL-8 or IL-18 in the BPH group.

**Conclusion:**

Omentin-1 may suppress the development of BPH and Lower serum omentin-1 levels in BPH patients might associated with higher prostate volume and higher IL-8 and IL-18 expression levels in their prostatic cells.

## Backgroud

Benign prostatic hyperplasia (BPH) is the most common urological disease in elderly men worldwide. BPH causes prostate enlargement and is associated with bladder outlet obstruction and various lower urinary tract symptoms (LUTS) [1]. BPH can also cause the deterioration of urinary function and quality of life and increase the risk of both urinary tract infection (UTI) and acute urinary retention, which requires surgical attention. Moreover, the medical costs for managing BPH are high, despite the fact that many men with BPH remained underdiagnosed and undertreated.

Although the mechanisms are not fully clear, metabolic status, inflammation, and hormonal status may contribute to the pathogenesis of BPH. Previous studies demonstrated evidence of strong correlations between metabolic syndrome and chronic inflammation in BPH pathogenesis and progression [2].

Omentin was primarily identified from cDNA library of human omental adipose tissue [3]. Omentin-1 (Gene name ITLN1), which is an isoform of omentin, shows its abundance in human plasma. Decreased serum omentin-1 levels are detected in patients with endocrine diseases such as type2 diabetes and obesity [4]. Meanwhile, it was proved that reduced omentin-1 level plays pivotal role in the causation of inflammatory diseases [5].

Previous studies on the association of BPH and adipokines (e.g., C-peptide, leptin and adiponectin) have reported conflicting results [6–8]. To the best of our knowledge, there are no reports of a relationship between omentin and BPH. Therefore, this matched case-control study investigated the serum omentin concentrations in patients with BPH.

## Methods

### Subjects

This study was approved by the Ethics Committee of the Second Xiangya Hospital of Central South University. A total of 70 males seen from January 2017 to January 2018 gave informed consent before participating in this study. Forty patients diagnosed with BPH who needed transurethral resection of the prostate (TURP) and thirty healthy males matched by age were assigned into two groups: the BPH group and the control group (CG). BPH was diagnosed based on clinical findings, rectal examination, ultrasound and confirmed by histopathological results. The healthy males were defined by ultrasound and International Prostate Symptom Score (IPSS). The prostate size of CG measured by ultrasound is less than 4 cm × 3 cm × 2 cm and IPSS is less than 7. The PSA values of all these men were less than 4 ng/mL.

### Methods

The anthropometric measurements obtained in this study included height, weight, systolic blood pressure (SBP), diastolic blood pressure (DBP), and waist circumference (WC). Body mass index (BMI) was calculated as the body weight divided by the height squared (kg/m2). We measured the WC at the umbilicus level. Biochemical parameters were measured in venous blood samples taken after at least a 10-h fast. Fasting blood samples were obtained to analyze blood urea nitrogen (BUN), triglyceride (TG), total cholesterol (TC), high-density lipoprotein (HDL), creatinine, prostate-specific antigen (PSA) and glucose levels using standard methods. The estimated glomerular filtration rates (eGFRs) were assessed using the following equation: eGFR = α × (SC÷0.9)-β × 0.993age [Note: SC is the serum creatinine level in mg/ml. α is 128, and β is 0.015 for SC ≤ 0.9 mg/dl or 79.56 μmol/l; otherwise, α is 119, and β is 0.688]. Prostate size was measured by ultrasound and the prostate volume (PV) was calculated from transverse images by using the prolate-ellipsoid formula: 0.524 × height×width×length [9].

### Omentin-1 measurement

Venous blood was collected after fasting followed with centrifugation of 4000 rpm at room temperature. Afterward, enzyme-linked immunosorbent assay (ELISA) was performed to determine concentration of omentin-1 in human serum (Apotech, Inc.).

Quantification of gene expression in BPH tissues by quantitative real-time PCR.

Total RNA was extracted using an RNAsimple Total RNA Kit (TIANGEN Biotech) and miRcute miRNA Isolation Kit (TIANGEN Biotech) and reverse transcribed using a RevertAid First Strand cDNA Synthesis Kit (Thermo Fisher). Quantitative RT-PCR was performed using SuperReal PreMix Plus (TIANGEN Biotech) and the following primers:

IL-8:

Fwd: CCAGGAAGAAACCACCGGA

Rev: GAAATCAGGAAGGCTGCCAAG

IL-18:

Fwd: AAAACCTGGAATCAGATTACTTTGG

Rev: TCCGGGGTGCATTATCTCTA

β-actin:

Fwd: TTCCTTCCTGGGCATGGAGTC

Rev: TCTTCATTGTGCTGGGTGCC

Specific gene expression was quantified using the 2-ΔΔCT method. Gene expression normalization was performed using β-actin as a reference gene.

### Statistical analysis

SPSS 22.0 software was used for the statistical analyses. The results are presented as the mean ± standard deviation (SD). Student’s t-test was used to determine the significance between two groups. Spearman’s rank correlation coefficient analyses (r) and the coefficient of determination (r^2^) were conducted to test for associations between omentin-1 levels and general clinical characteristics, biochemical parameters and relative gene expression. The influential factors for omentin-1 levels were identified using multiple-factor binary logistic regression analysis for meaningful clinical characteristics and biochemical parameters. Finally, the influential factors for BPH development were identified using multiple-factor binary logistic regression analysis for meaningful clinical characteristics and biochemical parameters. *P* < 0.05 was considered statistically significant, * indicates *p* < 0.05, ** indicates *p* < 0.01, *** indicates *p* < 0.001.

## Results

**Fig. 1:**
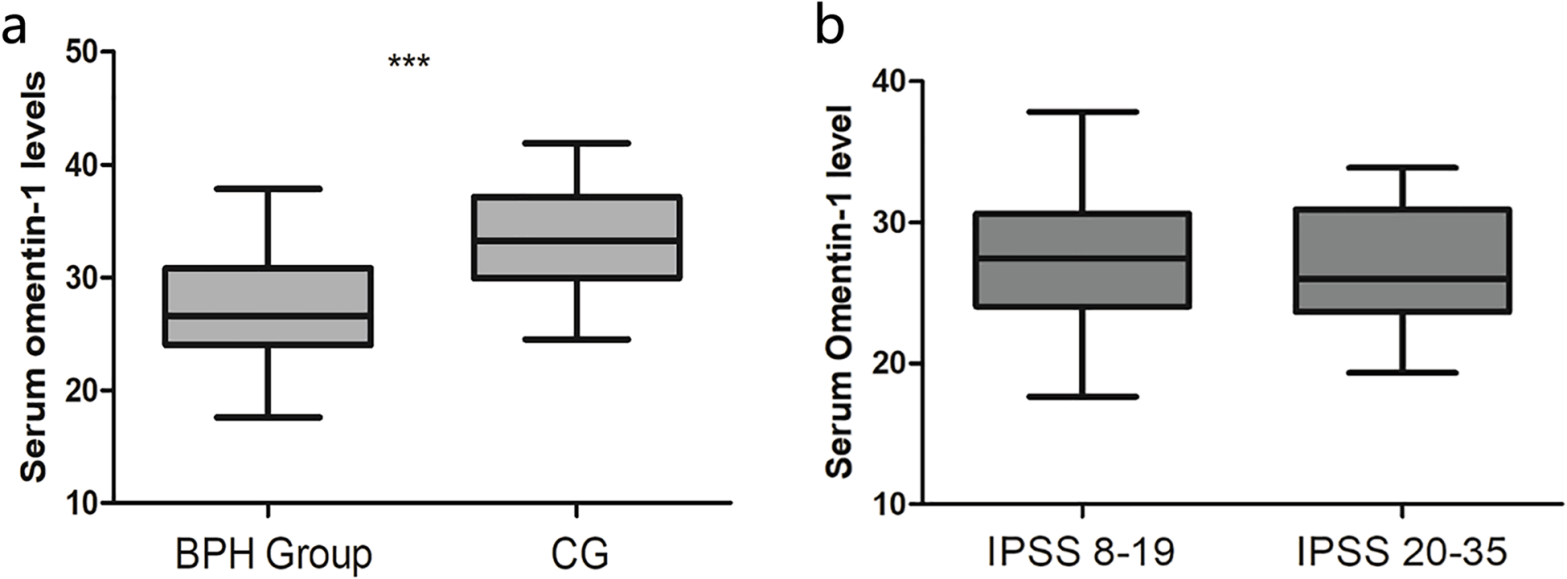
Serum omentin-1 levels. **a**. Serum omentin-1 levels in the BPH groups and CG. The concentrations of omentin-1 in human serum were determined using an enzyme-linked immunosorbent assay (ELISA) kit. **b**. The serum omentin-1 level in different IPSS level in BPH group. The BPH patients have been divided in two group according to the IPSS level. IPSS 8–19 indicates Level 2. IPSS 20–35 indicates level 3. The Vertical histograms show means ± SD

The general characteristics and biochemical parameters of the two groups are listed in Table 1. There were no statistically significant differences in age, WC, fasting glucose, SBP, DBP, TG, TC, HDL, fasting glucose, BUN and eGFR between the two groups. BMI, BUN, creatinine, PSA levels were significantly higher in the BPH group than in the CG. The omentin-1 levels were lower in the BPH group than in the CG (Fig. 1a).

**Table 1:** Comparison of the general characteristics and biochemical parameters

	BPH (*n* = 40)	CG (*n* = 30)	p
Age (years)	59.15 ± 7.382	59.07 ± 7.593	0.963
BMI (kg/m^2^)	26.20 ± 3.48	22.56 ± 3.01	< **0.001**
SBP (mmHg)	126.6 ± 10.8	124.5 ± 11.8	0.706
DBP (mmHg)	76.1 ± 8.3	72 ± 9.2	0.059
Waist circumference (cm)	90.33 ± 14.06	88.93 ± 12.89	0.671
TG (mmol/L)	1.81 ± 0.26	1.71 ± 0.18	0.091
TC (mmol/L)	4.56 ± 0.86	4.14 ± 1.01	0.073
HDL (mmol/L)	1.22 ± 0.31	1.18 ± 0.24	0.561
Fasting glucose (mmol/L)	4.76 ± 0.96	4.34 ± 0.94	0.076
BUN (mmol/L)	5.40 ± 1.57	4.80 ± 1.12	0.084
eGFR (mL/min/1.73 m^2^)	76.18 ± 8.37	79.97 ± 7.64	0.055
Creatinine (μmol/L)	81.46 ± 17.26	72.92 ± 17.63	**0.047**
PSA (ng/mL)	2.29 ± 0.89	1.72 ± 0.73	**0.005**
Omentin-1 (ng/mL)	27.31 ± 4.56	33.47 ± 4.92	< **0.001**
Prostate volume (mL)	62.00 ± 30.26	10.64 ± 1.29	< **0.001**

Multiple-factor binary regression analysis was performed to reveal the risk factors for BPH development from various meaningful clinical characteristics and biochemical parameters, including BMI, creatinine, PSA and omentin-1 (Table 2). Among these data, BMI and omentin-1 level were significantly associated with the BPH development.

**Table 2:** Multiple-factor binary logistic regression analysis

Variables	B value	SE value	OR value	95%CI	*P* value
BMI (kg/m^2^)	0.242	0.120	1.274	1.008–1.611	**0.043**
PSA (ng/mL)	0.457	0.390	1.579	0.720–3.395	0.242
Creatinine (μmol/L)	0.036	0.019	1.036	0.998–1.077	0.065
Omentin-1 (ng/mL)	−0.206	0.077	0.813	0.699–0.946	**0.008**

*SE* standard error, *OR* odds ratio, *CI* confidence interval

Correlations between the serum omentin-1 level and the biochemical properties in the BPH, CG and whole group were determined with Spearman’s rank correlation coefficient analysis. It was suggestive that serum omentin-1 levels have little correlation with age, SBP, DBP, WC, TG,TC,HDL, fasting glucose, BUN, eGFR or creatinine while negative correlations were found between serum omentin-1 levels and BMI in the BPH group (r = − 0.391, *p* = 0.013) as well as the whole group (r = − 0.457, *p* < 0.001) (Supplementary Table 1).

**Fig. 2:**
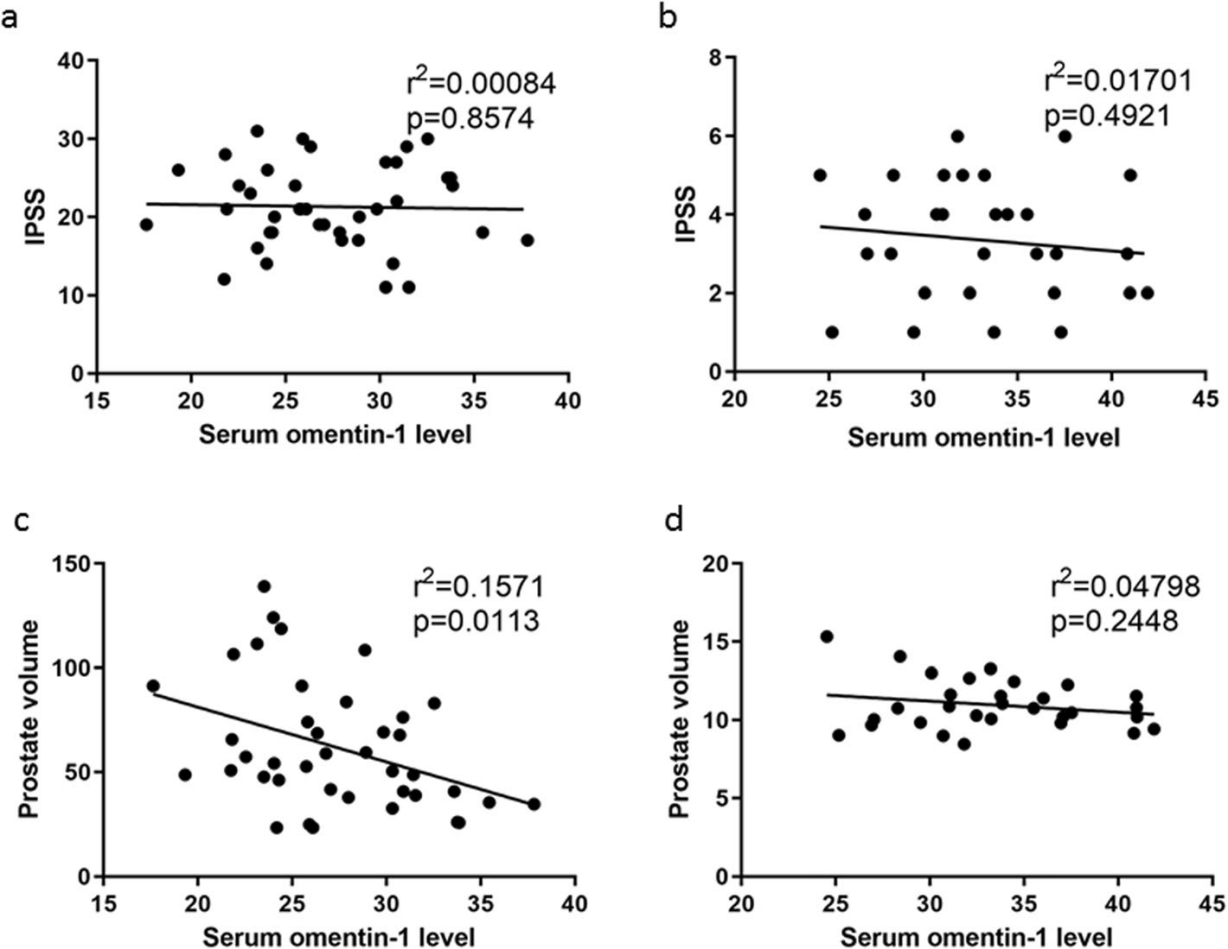
The correlation between serum omentin-1 and IPSS/Prostate volume. **a**. The coefficient of determination for IPSS and serum omentin-1 level in BPH group was established. **b**. The coefficient of determination for for IPSS and serum omentin-1 level in CG was established. **c**. The coefficient of determination for prostate volume and serum omentin-1 level in BPH group was established. **d**. The coefficient of determination for prostate volume and serum omentin-1 level in CG was established. The trend line is shown

The BPH patients have been divided in two group according to the IPSS level, IPSS 8–19 and IPSS 20–35. The serum omentin-1 level in these two group showed no significantly difference (Fig. 1b). And also, there was no significant correlation between serum omentin-1 and IPSS in either BPH group or CG (Fig. 2a, b). Alternative, serum omentin-1 level showed significant negative correlations with prostate volume in BPH group (Supplementary Table 2, Fig. 2c, d).

**Fig. 3:**
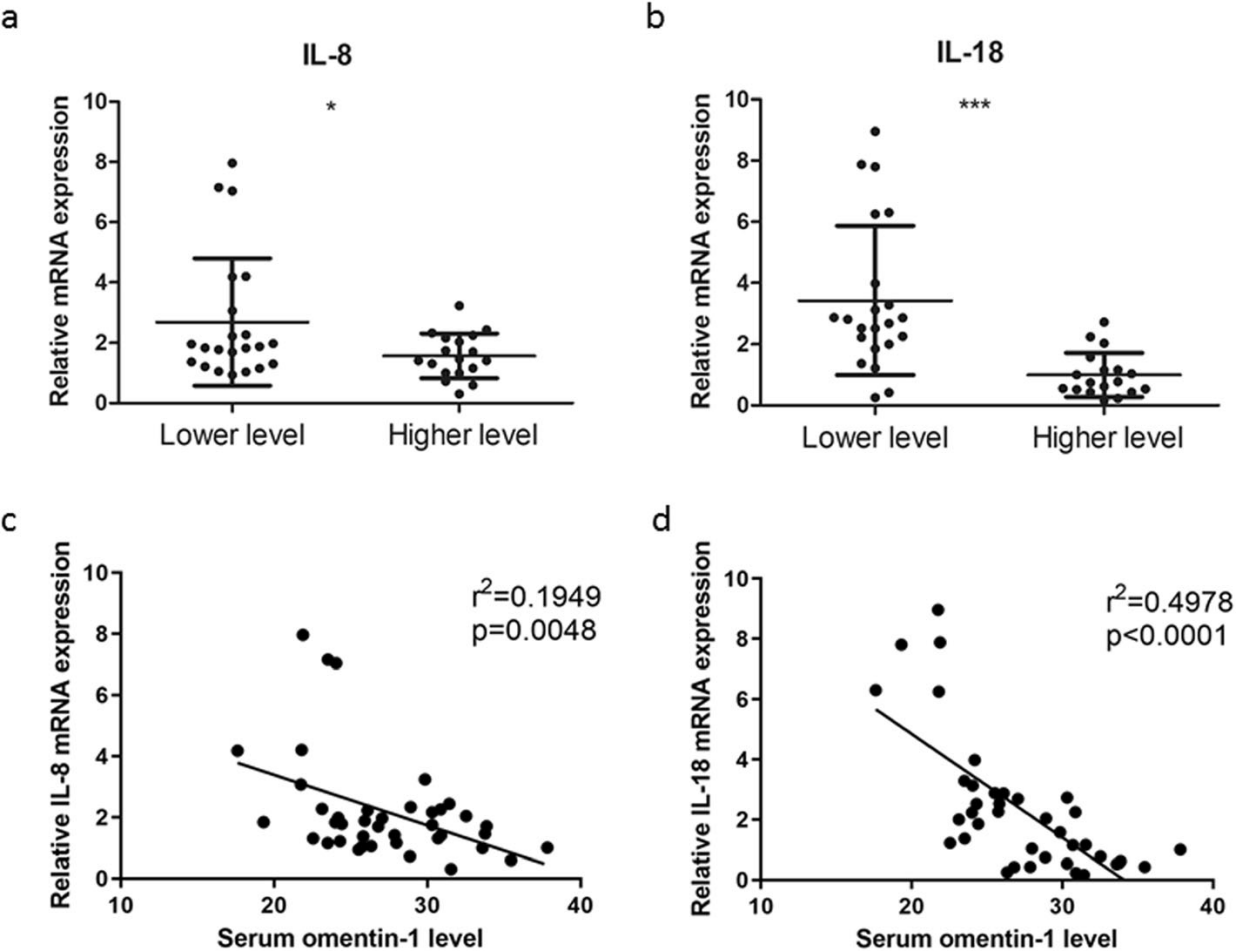
Relative gene mRNA expression according to omentin-1 Levels and their correlation. **a**, **b**. IL-8 and IL-18 mRNA expressions in clinical BPH specimen were determined by qRT-PCR. BPH patients were divided into two groups based on their serum omentin-1 levels. Results are presented as means ± SD. Specific gene expression was quantified using the 2-ΔΔCT method. Gene expression normalization was performed using β-actin as a reference gene. **c**. The coefficient of determination for IL-8 mRNA expression and serum omentin-1 level was established. **d**. The coefficient of determination for IL-18 mRNA expression and serum omentin-1 level was established. The trend line is shown

Interleukin-8 (IL-8) and interleukin-18 (IL-18) are two cytokines secreted mainly by prostatic cells and functionally related to Omentin-1 (Supplementary Figure 1) [10–13]. It has been shown that the mRNA expression of these two cytokines was significantly higher in BPH patients with LUTS [11–13]. Thus, in this study, we also checked the expression levels of IL-8 and IL-18 in our BPH patient samples. We found that the mRNA expression levels of IL-8 and IL-18 were significantly higher in the low serum omentin-1 group (levels lower than the median) (Fig. 3a, b). Moreover, there were significant negative correlations between serum omentin-1 levels and IL-8 and IL-18 mRNA expression in the BPH group (Supplementary Table 2, Fig. 3c, d).

## Discussion

With the changing demographic profile and an increasingly aging population in almost all societies, it is inevitable that the prevalence of BPH will increase.

Obesity is often comorbid with BPH. Body weight, BMI, and WC have been reported to be positively associated with prostate volume. Moreover, obesity was also associated with an increased risk of symptomatic BPH in several different international studies [14, 15]. In the present study, WC was greater in patients in the BPH group than those in the CG, but the difference was not statistically significant. BMI was significantly greater for the patients in the BPH group than for those in the CG, which suggests that increased body weight could be a factor in the etiology of BPH.

Several studies were done to examine the influence of diabetes using more objective measures of BPH, specifically prostate gland size. Increased prostate size and BPH surgery have strong association with the diagnosis of diabetes and increased serum insulin fasting plasma glucose level [16, 17]. In our study, glucose levels were not statistically significantly higher in the BPH group.

It is likely that inflammation plays a role in the development and progression of BPH [1, 18] because inflammatory cytokines are overexpressed in BPH tissues [19]. It remains unclear how inflammation mediates BPH. Obesity is considered a hormone-regulated disease when the secretion of pro-inflammatory cytokines and chemokine can promote the enlargement of adipocytes and at the mean time, influence prostate health [20]. These active hormones are known as adipokines, such as adiponectin. High adiponectin concentrations were associated with a reduced risk of symptomatic BPH [6].

Omentin is an anti-inflammatory adipokine in human with a higher expression in visceral adipose tissue than those found subcutaneously [21]. Omentin-1 is the major circulating form of omentin. Omentin showed a strong negative correlation with the incidence of inflammation, diabetes, obesity and metabolic syndrome [4], which all contribute to BPH progression. Decreased omentin-1 levels may play a role in the development of insulin resistance, type 2 diabetes mellitus and particularly obesity [22]. Through homeostasis model assessment, it was demonstrated that omentin-1 level is negatively correlated with BMI, WC and insulin resistance while it is positively associated HDL and adiponectin level [23]. A previous study showed that omentin-1 could downregulate pro-inflammatory cytokines to prevent inflammation-induced osteoporosis [5]. Di has reported that omentin suppresses pulmonary inflammation and promotes the endothelial barrier to prevent lipopolysaccharide (LPS)-induced acute respiratory distress syndrome (ARDS) [24]. Yao found that during prostatic hyperplasia, LPS/Toll-like receptor 4 signaling could upregulate transforming growth factor-β (TGF-β), which plays a role in the pathogenesis of BPH [25, 26]. Patients diagnosed with inflammatory bowel diseases such as Crohn’s disease or ulcerative colitis have lower level of circulating omentin-1 [27]. Uyeturk reported that serum omentin-1 levels were significantly higher in prostate cancer (PCa) patients than in BPH patients [28]. However, serum omentin-1 levels have not been previously examined in BPH patients and healthy people. In our present study, omentin-1 levels were dysregulated in patients in the BPH group compared to those in the CG. PSA levels were significantly different between the two groups but were negatively correlated with omentin-1 in the whole group. In addition, serum omentin-1 levels were negatively correlated with BMI in the BPH group and in the whole group. Serum omentin-1 level also showed significant negative correlation with prostate volume in BPH group. Furthermore, we found that omentin-1 might be a protective factor of BPH development.

In patients with acute myocardial infarction, omentin-1 negatively correlated with IL-18, and IL-18 remained an independent determinant of omentin-1 levels [29]. In research on obese people who participated in training experiments, serum omentin-1 levels decreased significantly, and IL-18 levels increased significantly after detraining [30]. These studies showed that serum omentin-1 levels may negatively correlate with IL-18 in BPH tissues. IL-18 could be secreted by prostatic epithelial cells and respond to several inflammatory stimuli [31]. IL-18 itself directly influences prostatic stromal cell proliferation [12]. Similarly, our results suggested that in the BPH group, serum omentin-1 levels were significantly negatively correlated with IL-18 expression in prostatic cells. IL-8, produced by BPH prostate epithelial cells as a reliable biomarker of inflammation in BPH, can promote the proliferation of non-senescent epithelial and stromal cells and contributes to the increased tissue growth in BPH [11, 13]. IL-8 expression was also significantly negatively correlated with serum omentin-1 levels in our investigation, which supports the findings that omentin-1 could reduce IL-8 production in kidney tissue [32]. From all these data, what can be hypothesized is that men with higher BMI might show lower serum omentin-1 level, thereby contribute to the development of BPH via facilitating the activation of prostatic inflammation.

## Conclusion

In summary, we found that serum omentin-1 levels were decreased in patients with BPH. Omentin-1 may suppress the development of BPH and Lower serum omentin-1 levels in BPH patients might associated with higher prostate volume and higher IL-8 and IL-18 expression levels in their prostatic cells. Further studies establishing the mechanism underlying this phenomenon would allow better management of BPH development.

## Supplementary information

**Additional file 1: Figure S1**. Functional protein association networks of Omentin-1 (ITLN1). The functional protein association networks of Omentin-1 (Gene name: ITLN1) was shown. Cytokines such as Interleukin-18 (IL18) were reported to be closely linked to ITLN1.

**Additional file 2: Table S1**. Spearman’s rank correlation coefficient analysis of serum omentin-1 levels with the general clinical characteristics and biochemical parameters.

**Additional file 3: Table S2**. Spearman’s rank correlation coefficient analysis of serum omentin-1 levels with the general clinical characteristics and biochemical parameters.

## Data Availability

The datasets used and/or analysed during the current study are available from the corresponding author on reasonable request.

## Abbreviations

BPH: benign prostatic hyperplasia
BMI: Body mass index
SBP: Systolic blood pressure
DBP: Diastolic blood pressure
WC: Waist circumference
BUN: Blood urea nitrogen
TG: Triglyceride
TC: Total cholesterol
HDL: High-density lipoprotein
PSA: Prostate-specific antigen
eGFRs: Estimated glomerular filtration rates
PV: Prostate volume
CG: Control group
IL-8: Interleukin-8
IL-18: Interleukin-18
