# Supplementary figures and images for: Serum omentin-1 level in patients with benign prostatic hyperplasia
Source: BMC Urol. 2020 May 6;20:52. doi: 10.1186/s12894-020-00623-4 (PMC7203873; doi:10.1186/s12894-020-00623-4)

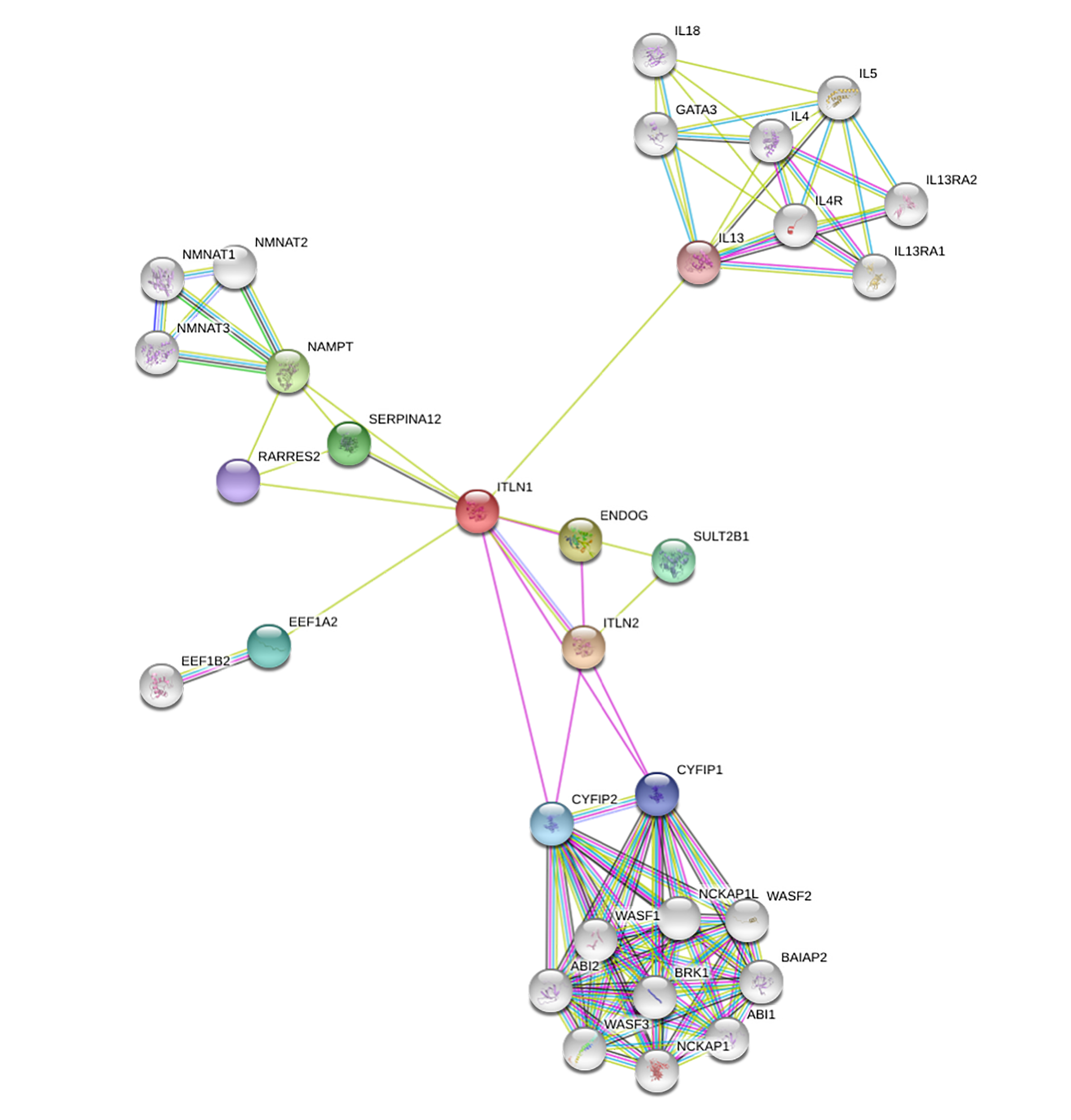

Supplement: Supplementary file 1 — Additional file 1: Figure S1. Functional protein association networks of Omentin-1 (ITLN1). The functional protein association networks of Omentin-1 (Gene name: ITLN1) was shown. Cytokines such as Interleukin-18 (IL18) were reported to be closely linked to ITLN1. [file 12894_2020_623_MOESM1_ESM.tif]
