# Supplementary material for: Serum omentin-1 level in patients with benign prostatic hyperplasia
Source: BMC Urol. 2020 May 6;20:52. doi: 10.1186/s12894-020-00623-4 (PMC7203873; doi:10.1186/s12894-020-00623-4)
Supplement: Supplementary file 2 — Additional file 2: Table S1. Spearman’s rank correlation coefficient analysis of serum omentin-1 levels with the general clinical characteristics and biochemical parameters. [file 12894_2020_623_MOESM2_ESM.docx]

**Supplementary Table 1. Spearman’s rank correlation coefficient analysis of serum omentin-1 levels with the general clinical characteristics and biochemical parameters**

| Variables | BPH Group | |  | CG | |  | Whole Group | |
| --- | --- | --- | --- | --- | --- | --- | --- | --- |
|  | r | p |  | r | p |  | r | p |
| Age (years) | 0.029 | 0.859 |  | -0.217 | 0.249 |  | -0.067 | 0.581 |
| BMI (kg/m^2^) | -0.391 | **0.013** |  | -0.284 | 0.128 |  | -0.544 | <**0.001** |
| SBP (mmHg) | 0.071 | 0.662 |  | -0.001 | 0.995 |  | 0.046 | 0.705 |
| DBP (mmHg) | 0.226 | 0.161 |  | 0.058 | 0.759 |  | 0.058 | 0.632 |
| Waist circumference (cm) | -0.112 | 0.49 |  | -0.175 | 0.355 |  | -0.164 | 0.176 |
| TG (mmol/L) | -0.015 | 0.928 |  | -0.266 | 0.155 |  | -0.199 | 0.098 |
| TC (mmol/L) | 0.284 | 0.076 |  | 0.273 | 0.144 |  | 0.139 | 0.252 |
| HDL (mmol/L) | 0.032 | 0.844 |  | -0.337 | 0.068 |  | -0.121 | 0.316 |
| Fasting glucose (mmol/L) | 0.132 | 0.418 |  | 0.219 | 0.245 |  | 0.075 | 0.537 |
| BUN (mmol/L) | 0.179 | 0.269 |  | -0.037 | 0.847 |  | -0.019 | 0.873 |
| Creatinine (µmol/L) | -0.049 | 0.763 |  | 0.099 | 0.604 |  | -0.095 | 0.434 |
| eGFR (mL/min/1.73 m^2^) | 0.106 | 0.517 |  | -0.046 | 0.809 |  | 0.145 | 0.231 |
| PSA (ng/mL) | -0.306 | 0.055 |  | -0.299 | 0.108 |  | -0.373 | **0.001** |
