# Supplementary material for: Serum omentin-1 level in patients with benign prostatic hyperplasia
Source: BMC Urol. 2020 May 6;20:52. doi: 10.1186/s12894-020-00623-4 (PMC7203873; doi:10.1186/s12894-020-00623-4)
Supplement: Supplementary file 3 — Additional file 3: Table S2. Spearman’s rank correlation coefficient analysis of serum omentin-1 levels with the general clinical characteristics and biochemical parameters. [file 12894_2020_623_MOESM3_ESM.docx]

**Supplementary Table 2. Spearman’s rank correlation coefficient analysis of serum omentin-1 levels with the general clinical characteristics and biochemical parameters**

| Variables | BPH Group | |  | CG | |  |
| --- | --- | --- | --- | --- | --- | --- |
|  | r | p |  | r | p |  |
| IPSS | -0.02933 | 0.8574 |  | -0.1304 | 0.4921 |  |
| Prostate volumn(ml) | -0.3963 | **0.0113** |  | -0.2190 | 0.2448 |  |
| IL-8 | -0.403 | **0.0099** |  | N/A | N/A |  |
| IL-18 | -0.7255 | **<0.0001** |  | N/A | N/A |  |
